# Technical Note: Practical implementation strategies of cycloidal computed tomography

**DOI:** 10.1002/mp.14821

**Published:** 2021-06-24

**Authors:** Oriol Roche i Morgó, Fabio Vittoria, Marco Endrizzi, Alessandro Olivo, Charlotte K. Hagen

**Affiliations:** ^1^ Department of Medical Physics and Biomedical Engineering University College London London WC1E 6BT UK; ^2^ ENEA Radiation Protection Institute Bologna Italy

**Keywords:** cycloidal computed tomography, micro computed tomography, phase contrast, spatial resolution, structured illumination

## Abstract

**Purpose:**

Cycloidal computed tomography is a novel imaging concept which combines a highly structured x‐ray beam, offset lateral under‐sampling, and mathematical data recovery to obtain high‐resolution images efficiently and flexibly, even with relatively large source focal spots and detector pixels. The method reduces scanning time and, potentially, delivered dose compared to other sampling schemes. This study aims to present and discuss several implementation strategies for cycloidal computed tomography (CT) in order to increase its ease of use and facilitate uptake within the imaging community.

**Methods:**

The different implementation strategies presented are step‐and‐shoot, continuous unidirectional, continuous back‐and‐forth, and continuous pixel‐wise scanning. In step‐and‐shoot scans the sample remains stationary while frames are acquired, whereas in all other cases the sample moves through the scanner continuously. The difference between the continuous approaches is the trajectory by which the sample moves within the field of view.

**Results:**

All four implementation strategies are compatible with a standard table‐top x‐ray setup. With the experimental setup applied here, step‐and‐shoot acquisitions yield the best spatial resolution (around 30 µm), but are the most time‐consuming (1.4 h). Continuous unidirectional and back‐and‐forth images have resolution between 30 and 40 µm, and are faster (35 min). Continuous pixel‐wise images are equally time‐efficient, although technical challenges caused a small loss in image quality with a resolution of about 50 µm.

**Conclusion:**

The authors show that cycloidal CT can be implemented in a variety of ways with high quality results. They believe this posits cycloidal CT as a powerful imaging alternative to more time‐consuming and less flexible methods in the field.

## 1 INTRODUCTION

X‐ray micro computed tomography (CT)^1^, ^2^ is a powerful technique for nondestructive, high‐resolution imaging, with applications in clinical and preclinical contexts. The achievable spatial resolution is defined by the overall system blur obtained from the convolution of the source focal spot and the detector pixel point spread function, projected onto the same plane.^3^ The distances between the source, sample, and detector also affect the spatial resolution due to magnification; however, the maximum achievable resolution is determined by the source and detector properties. High resolutions can be achieved using detectors with a small pixel size, but these often impose limited fields of view. Flexibility in adapting to the resolution needs of the sample is also lacking, since hardware with that purpose is available (e.g., sources with variable focal spots^4^) but its use is not well‐spread. The resolution could be changed by varying the distances between the elements of the system, but this again comes at a cost of the field of view.^5^ Under these conditions, multiscale, high resolution imaging is constrained by the available setup.

These shortcomings can be overcome with cycloidal computed tomography.^6^, ^7^ In cycloidal CT, the x‐ray beam is structured into narrow beamlets a few to tens of micrometers wide which, with an appropriate sampling scheme, allow accessing higher spatial resolution than usually achievable with a given source and detector. Previous structured illumination techniques have utilized similar setups but require a dense sampling scheme called “dithering,” by which the sample is translated laterally in multiple steps per rotation angle in order to ensure that it is fully illuminated.^8^, ^9^ Dithering introduces dead times in the acquisition process, thus making it lengthy and inefficient, as well as incompatible with continuous acquisitions.

Instead, cycloidal CT consists of *simultaneously* rotating and moving the sample laterally. With this motion, the images are under‐sampled because the x‐rays do not cover the whole sample at every projection, but this is compensated for with an appropriate data recovery method. Fewer frames are acquired compared to dithering, significantly reducing the scan time and, potentially, the delivered dose, making it more efficient especially when applied continuously. It is also a highly flexible concept since it can be implemented both in attenuation and phase contrast modes.

Cycloidal CT has only recently been introduced, and its characteristics offer the flexibility to implement it with different acquisition strategies. In this study we have explored four different strategies and compared them in terms of their efficiency and image quality.

## 2 MATERIALS AND METHODS

### 2.A Cycloidal computed tomography

**Fig. 1:**
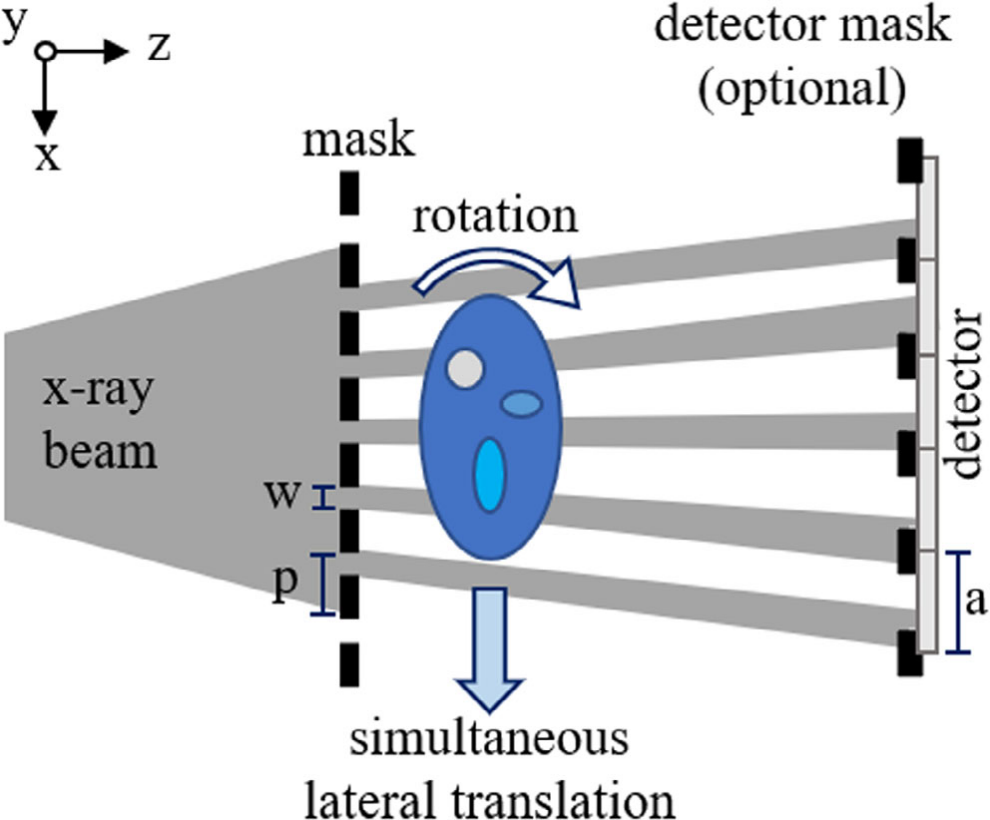
Schematic representation of the cycloidal imaging setup. By simultaneously rotating and translating the sample, we can obtain cycloidally sampled datasets. The first mask splits the beam into beamlets. The second (optional) mask transforms the setup into an edge‐illumination phase contrast system.

In order to access higher spatial resolutions, cycloidal CT requires structuring the x‐ray beam into an array of beamlets. This is achieved with the help of an absorbing mask (“sample mask”) with periodically spaced slit‐shaped apertures, placed upstream of the sample and parallel to the detector. Usually, the distance between the mask and the sample is small enough to be considered negligible. The aperture width (*w*) of the mask must be smaller than the overall system blur to ensure that higher spatial frequencies are available.^8^ The mask period (*p*) must be large enough to avoid (or minimize) overlap of the beams, which blurs the high frequency signal from the sample. Usually, *p* matches the size (*a*) of the detector pixels when scaled to the isocenter of the sample. In cases where a larger period is needed to keep the beams separated, a “line‐skipping” mask can be employed, which has a period equivalent to two (or three, or four…) pixels. A schematic of the setup can be found in Fig. 1.

Under these conditions, cycloidal sampling can be employed. The sample rotates around the vertical (y‐) axis as it is translated across the field of view. A frame is acquired at a rotation angle and lateral position, then the sample is rotated and shifted, another frame is acquired, and so on until the scan is complete. In a continuous unidirectional scan, the trajectory of a feature in the sample is a cycloid; in all other cases the trajectory corresponds to pieces of cycloids. The distance *d* by which the sample is translated at each angular step is a fraction of the mask period *p*.

The cycloidal technique acquires only one frame per projection angle, in contrast with dithering, which requires several frames per angle to ensure that the whole sample is illuminated. By reducing the number of frames, the scanning time and dose can be decreased and efficiency improved.^7^ The exact dose‐saving capabilities of cycloidal CT are an ongoing area of research, but more information is available in Ref. [7].

**Fig. 2:**
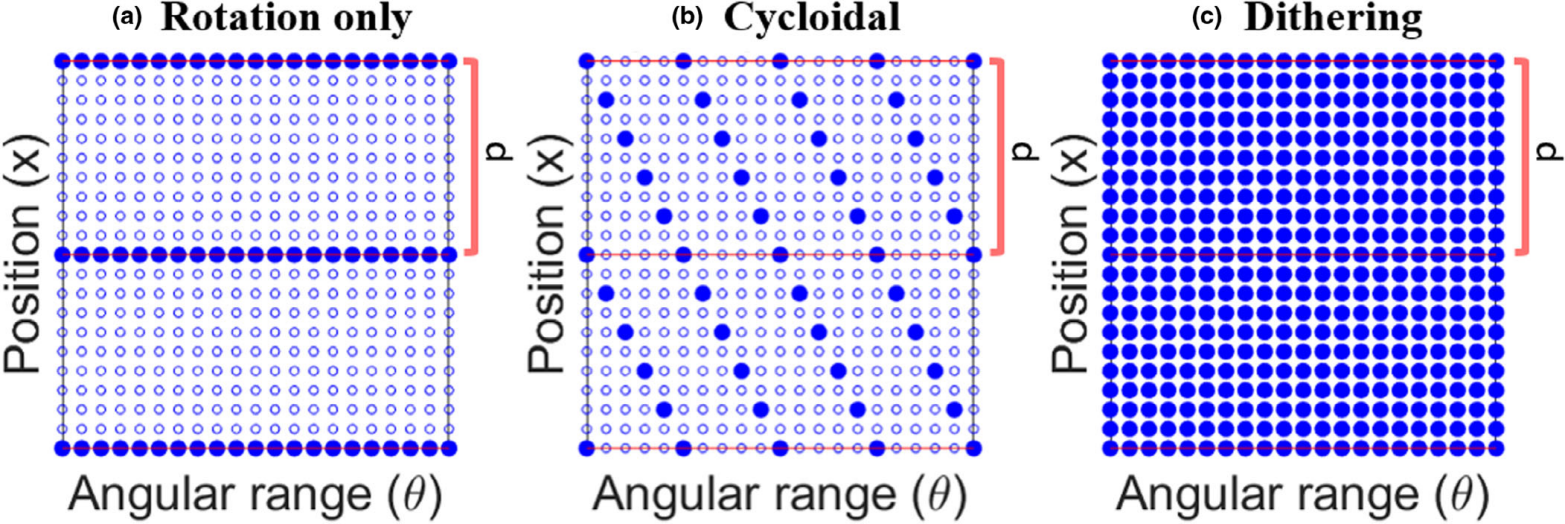
Sketch of the sinogram sampling grid shown for two mask periods and a subset of angles. The filled dots represent data acquired during a scan, while the empty dots represent data that are not acquired. Panel a shows the grid if the sample only rotates. Panel b shows the grid when applying a cycloidal scheme (with *d* *=* 0.2*p*). Panel c shows the fully sampled, dithered grid. The data points in panels a and b are the same, but they are more evenly distributed in the latter.

The cycloidal motion ensures that the data can be spread uniformly on the sinogram sampling grid. With no lateral motion (i.e., just rotation) the images are severely under‐sampled in the lateral direction [Fig. 2(a)]. In cycloidal sampling the images are also under‐sampled (the same number of frames is acquired, i.e., one per rotation angle), but the data are spread out on the grid [Fig. 2(b)]. The exact distribution depends on the value of *d* and the angular sampling step. A mathematical data recovery method is then used to make up for this under‐sampling. The method currently used is bicubic splines interpolation, which has been found to outperform simpler forms of interpolation (e.g., linear). In dithering, the sinogram is complete [Fig. 2(c)], which gives access to high spatial frequencies. With the appropriate value of *d*, the cycloidal technique can provide similarly high frequencies at a fraction of the frames.

### 2.B Edge‐illumination x‐ray phase contrast

The cycloidal imaging setup can be transformed into an edge‐illumination (EI) x‐ray phase contrast imaging setup. EI can be implemented in laboratory settings with conventional x‐ray sources.^10^ On top of the conventional attenuation of x rays, the method exploits the phase effects introduced by a sample, which can increase image contrast particularly in materials with little absorption, such as low‐Z biological soft tissues.^11^, ^12^

The EI setup requires adding a second absorbing mask (“detector mask”) with periodically slit‐shaped apertures in front of the detector, shielding parts of the pixels from the incoming beamlets. This mask is placed relative to the sample mask so that, without a sample, each x‐ray beamlet falls on the edge created by the detector mask apertures, giving a specific intensity signal.^13^, ^14^ This signal will change according to the refraction introduced by the sample, increasing or decreasing the intensity on the detector if the beamlets move further onto the pixels or the mask septa, respectively. These changes in intensity give rise to phase contrast.^13^, ^14^, ^15^, ^16^, ^17^

EI raw images contain a combination of attenuation and refraction effects. In order to reconstruct the phase map in CT, a process of “phase retrieval” is required, which isolates the phase signal. There are several approaches, but the “single‐shot” retrieval is particularly efficient because it only requires one image per projection angle,^18^, ^19^ as opposed to some other less efficient methods.^20^

### 2.C Practical implementation strategies

Four practical implementation strategies have been investigated. In all cases, the sample is rotated and translated simultaneously, but the exact form of the lateral translation changes.

#### 2.C.1 Step‐and‐shoot scanning

**Fig. 3:**
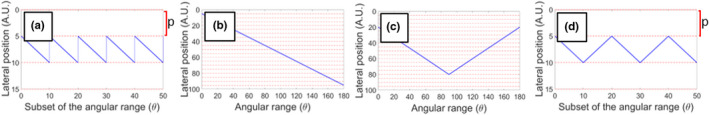
Schematics of the movement on the sinogram space for step‐and‐shoot (a), continuous unidirectional (b), back‐and‐forth (c), and pixel‐wise (d) scans. The solid line shows the movement across the field of view. The dashed lines indicate the pixel boundaries. Panels a and d show just a section of the field of view because the sample moves within one pixel.

The sample is stationary in every position as frames are acquired, and the movement from one position to the next is performed in between acquisitions. The sample moves laterally by *d* with every angular step, up to one mask period, then it is shifted back to its initial position and the same stepped movement is repeated until the scan is complete. The start and stop of the motors in between acquisitions introduces substantial dead times. A schematic is shown in Fig. 3(a).

#### 2.C.2 Continuous unidirectional scanning

**Fig. 4:**
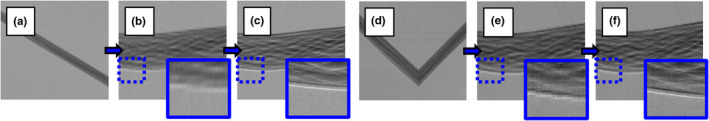
Example of the process of regridding with continuous unidirectional data (panels a–c) and continuous back‐and‐forth data (panels d–f). The first panels show the sinogram as it is acquired. The second show the same data after regridding, that is, after positioning the data points in what would be their pixel position in a step‐and‐shoot approach. Panels c and f show the sinogram after data recovery.

The sample is moved continuously, the rotation and lateral motion starting simultaneously. The lateral motion extends along one direction, from one side of the field of view toward the other side at a constant speed. Due to the periodicity of the pixel array, the sample is probed in the same locations as in step‐and‐shoot, but the data are acquired with different pixels. Because of this, the acquisition produces a slanted sinogram [Fig. 3(b)]. To reconstruct the image, “regridding” is required, where the data are repositioned into what would be their original pixel position (this process is illustrated in Fig. 4). The regridding is straightforward as long as the translational speed of the sample, and hence the value of *d*, remains constant throughout the scan.

#### 2.C.3 Continuous back‐and‐forth scanning

The sample movement is performed continuously; however, the lateral translation is performed back‐and‐forth between two points in the field of view, so the sample changes direction at least once during the scan [Fig. 3(c)]. A regridding process is required, but every time that the sample changes direction the translation motor decelerates and accelerates back to a constant speed and, while that happens, the value of *d* is not constant. If there are not many changes in direction, this may be considered negligible. Otherwise, the exact position of the sample while the motor changes speeds can be calculated using the acceleration profiles of the system, and the bicubic splines interpolation can be done on the corresponding irregular grid.

#### 2.C.4 Continuous pixel‐wise scanning

The sample continuously moves back‐and‐forth within one (de‐magnified) pixel [Fig. 3(d)]. Since it stays within its “initial” pixel during this strategy, there is no need for regridding. However, because of the short travel distance the motors do not reach a constant speed so the value of *d* is not constant. If the motors are accurate enough, the acceleration profiles can be used to find the position of the sample. These values are then used for interpolating on an irregular grid.

### 2.D Acquisition parameters

The four implementation strategies were tested on a phantom made of polyethylene spheres between 425 and 500 μm in diameter, contained in a plastic straw of 3 mm in diameter. Scans were acquired over 180º with an angular step of 0.2º and an exposure time of 2 s per frame. Nine hundred frames were acquired for all strategies. The sample was translated a total distance of 18.71352 mm in the unidirectional and back‐and‐forth cases; in the latter, this was split in two trips [see Fig. 3(c)]. Strictly, the scan geometry is fan beam, but our data were treated as parallel beam because the sample is small enough that the distortion from the fan angle remains largely below the spatial resolution.

Before testing the four strategies, tests were carried out to empirically determine the optimal value of *d*. Both simulated and experimental data suggest using *d = *0.25*p* for high‐quality results. Therefore, all scans were acquired using *d = *0.25*p* (at least nominally, since the value changes when the motors accelerate or decelerate).

As the sphere phantom is weakly attenuating, all data were acquired in x‐ray phase contrast mode, with the EI technique. The detector was a CMOS‐based flat panel C9732DK‐11 from Hamamatsu (Japan) with 50 µm pixels and a focal spot size of approximately 70 µm. A “line‐skipping” detector mask (with an aperture of 17 µm and a period of 98 µm) was employed to reduce the effects of pixel cross talk; this resulted in an effective pixel size of 80 µm (at the sample plane). The source was a MicroMax 007 HF x‐ray tube with a Molybdenum target from Rigaku (Japan), operating at 40 kVp and 20 mA. The sample mask had a 79 µm period and 10 µm apertures. It was placed approximately at 0.7 m from the source and 0.02 m from the sample. The distance between the source and the detector was 0.875 m. Both masks were fabricated by Creatv Microtec (USA) by electroplating gold onto a graphite substrate. The data processing included flat field and dark field corrections, and single‐shot phase retrieval.^18^, ^19^ Prior to the retrieval, the missing data in the sinograms were recovered with bicubic splines interpolation. CT images were reconstructed using filtered back projection with a Ram‐Lak filter, implemented with MATLAB’s iradon function.

## 3 RESULTS

**Fig. 5:**
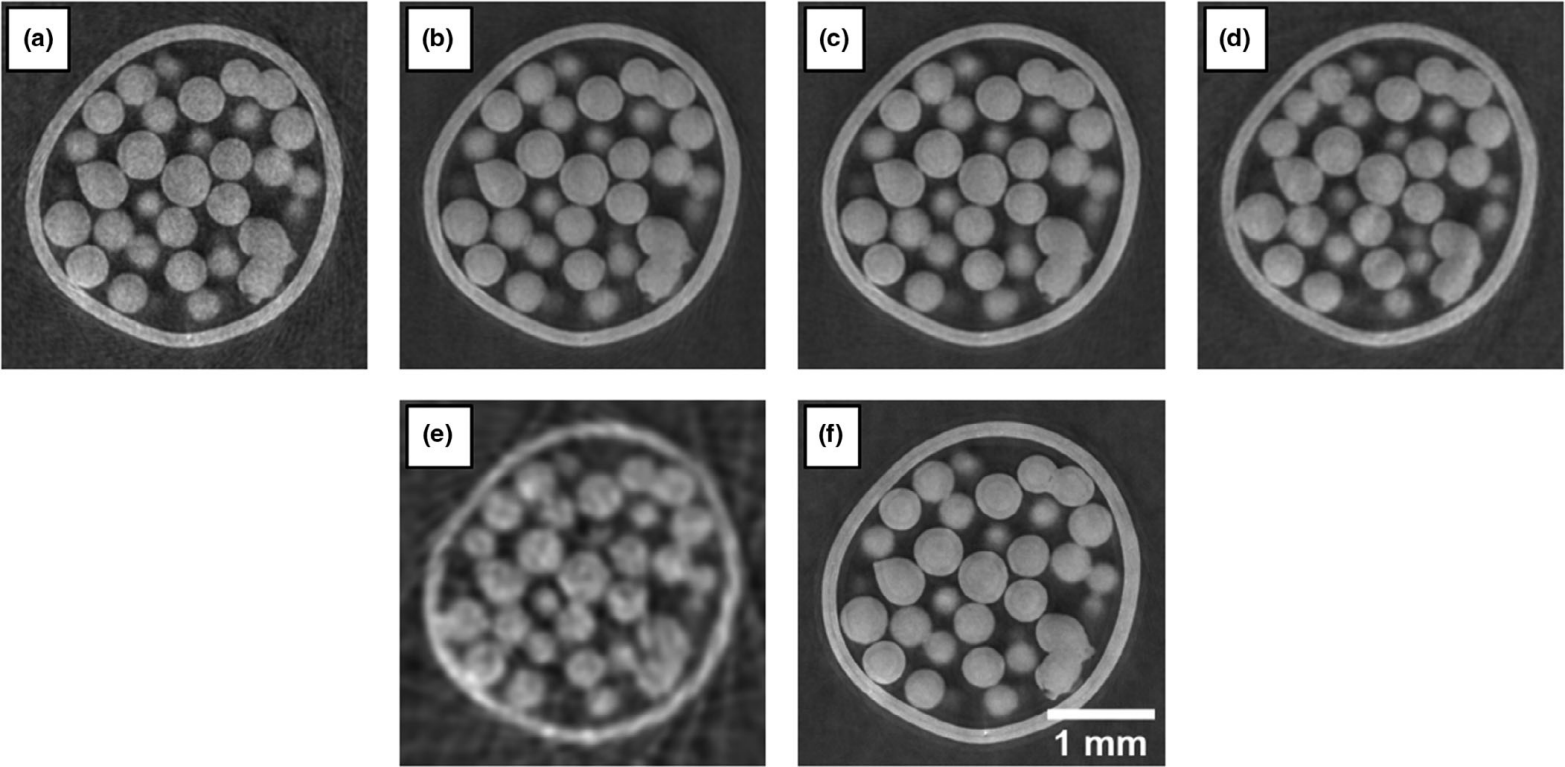
Reconstructed images of a polyethylene spheres phantom. The first four are cycloidal CT images: (a) step‐and‐shoot, (b) continuous unidirectional, (c) continuous back‐and‐forth, and (d) continuous pixel‐wise. Panel e shows a rotation only image and panel f shows a dithered image. The step‐and‐shoot and rotation only data were subsampled from the dithered data; in the rotation only case, the subsampling and posterior interpolating cause the apparent streak artifacts. Slight differences between the panels are due to the movement of the plastic spheres in between scans.

**Fig. 6:**
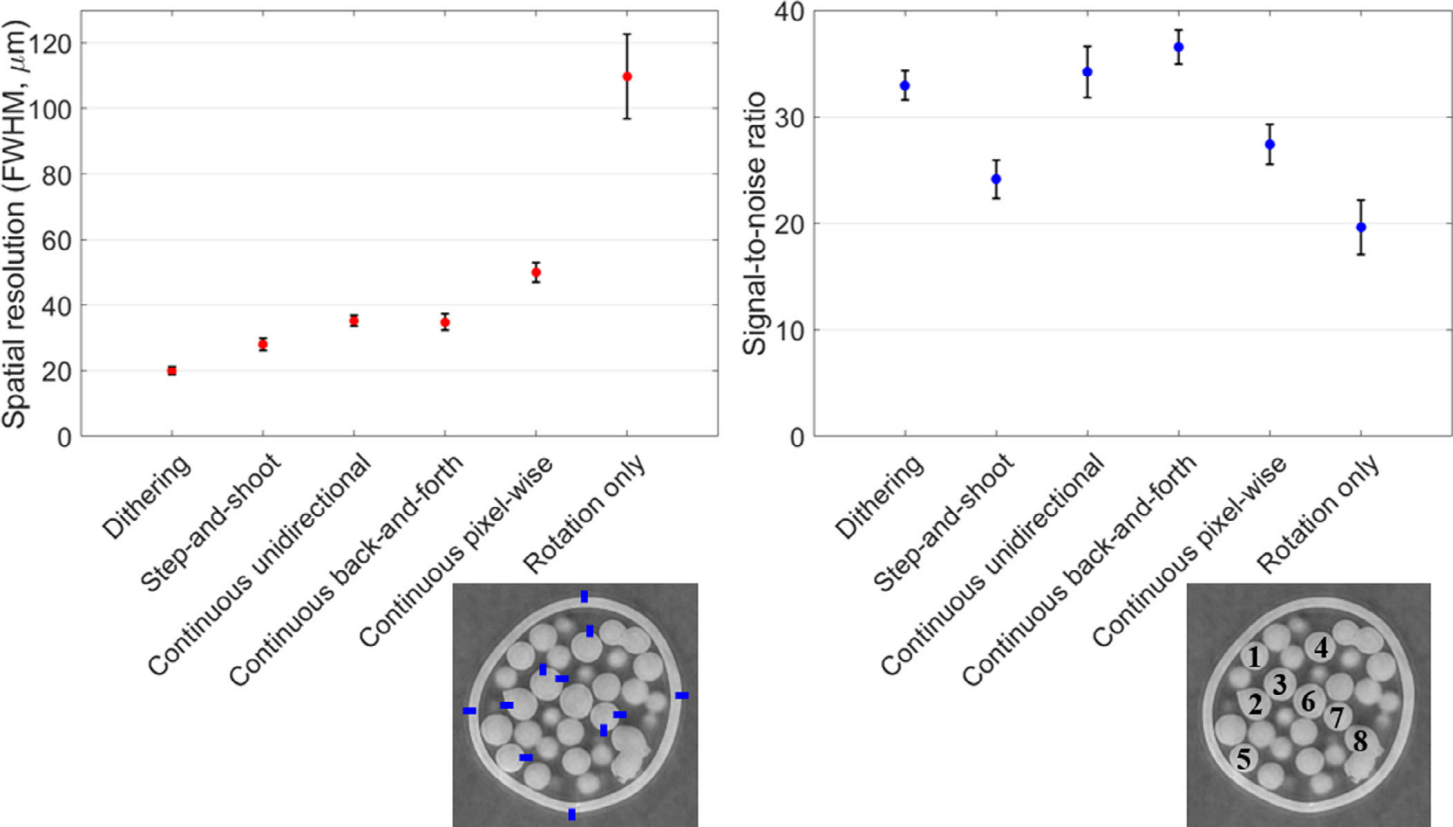
Image quality analysis for cycloidal CT implementations. Plot a shows the spatial resolution of cycloidal images compared to the gold standard. The image in the bottom shows where the resolution was measured. The final values are the mean of all measurements, and error bars are the standard error. Plot b shows the signal‐to‐noise ratio of cycloidal images, compared to dithered and rotation only cases. The image in the bottom shows where the measurements were taken. The final values are the average of all eight spheres, and error bars are the standard error.

The reconstructed images obtained from the four different strategies are displayed on Fig. 5. Panels a, b, c, and d show the step‐and‐shoot, continuous unidirectional, continuous back‐and‐forth, and continuous pixel‐wise results, respectively. These can be compared to the dithered dataset, taken as a gold standard, in panel f. The cycloidal strategies give clear images, comparable in quality to the dithered case. Only the pixel‐wise image appears somewhat blurrier. The cycloidal and dithered images can also be compared to the rotation only image in panel e, which is acquired with no lateral translation. The gain in spatial resolution, both from dithering and the cycloidal approaches, is evident. These observations are confirmed by the spatial resolution measurements [Fig. 6(a)], calculated as the full width at half maximum (FWHM) of the line spread function at the edge of the spheres. The dithered image features a spatial resolution of 20 ± 1 µm. The cycloidal CT images acquired with the step‐and‐shoot, continuous unidirectional, and continuous back‐and‐forth strategies have similar resolutions, between 20 and 40 µm, with step‐and‐shoot being slightly better than the other two. This agrees with previous results, where the in‐slice resolution of the step‐and‐shoot approach was found to be only slightly below that of the dithered image.^7^ Among the cycloidal CT images, the continuous pixel‐wise approach yields the lowest resolution around 50 µm.

The signal‐to‐noise ratio (SNR) of the images is displayed on Fig. 6(b). Dithered, unidirectional and back‐and‐forth images have similar SNR, above the step‐and‐shoot and pixel‐wise approaches. Rotation only scanning has the worst SNR, as can be visually confirmed from panel 5e.

For the continuous unidirectional and back‐and‐forth cases the data were corrected for a movement of a few pixels in the vertical (y‐) direction, since the translation stage was not perfectly horizontal. This was done by assuming a linear slope in the vertical translation and interpolating. There was also a misalignment between the continuous (unidirectional and back‐and‐forth) sinograms and the dithered counterparts, which arises from a systematic error in the motor speed. This was corrected by registering each continuous projection to its dithered equivalent.

The pixel‐wise motion was not uniform due to the inaccuracy of the motors. This was corrected manually by determining the approximate position of the sample for every projection. The use of servo motors, which have lower error in position, would eliminate this problem.

## 4 DISCUSSION

While all of the presented implementation strategies for cycloidal CT lead to images with a much higher spatial resolution than by simply rotating the sample, and some of which are comparable to the dithered images, each strategy exhibits advantages and disadvantages. Step‐and‐shoot scans are straightforward to implement because the data are acquired when the sample is static and it does not require any regridding. This is an advantage because the regridding process is error prone (as the speed of the translation motor has to be known precisely). However, the step‐and‐shoot approach requires starting and stopping the motors, which introduces dead times and lengthens the scan time. The continuous strategies avoid these dead times and are therefore around 2.5 times faster (or more, depending on how often flat fields in step‐and‐shoot are acquired). The scanning times are summarized in Table  I.

**Table I:** Scanning times (in minutes) for different techniques, assuming an exposure time of 2 s, 900 projections and 1 flat field every 30 projections for dithering and step‐and‐shoot cycloidal CT.

	Scanning time (min)
Dithering (eight dithering steps)	660
Cycloidal step‐and‐shoot	84
Cycloidal continuous unidirectional	35
Cycloidal continuous back‐and‐forth	35
Cycloidal continuous pixel‐wise	35

On top of this, the continuous unidirectional and back‐and‐forth approaches present better SNR than the step‐and‐shoot (and pixel‐wise) options, despite using the same number of frames. The reason for this is still unclear and it will be investigated as part of future work.

Between the continuous options, the unidirectional method is the easiest to implement because the sample movement is constant. However, because it is done in one direction, the field of view needs to be wide enough to include the whole trajectory. For small samples (a diameter around 5–10% of the field of view) this is feasible; but for larger samples it may be difficult to find an appropriate setup.

The back‐and‐forth alternative relaxes the field of view requirements. In our case, the assumption of a “constant *d*” at the point where the sample changes direction does not seem to affect the final result; however, the more times this assumption needs to be made (i.e., the more times the sample flips direction), the more the image quality will worsen. This is in fact what happens for the pixel‐wise case, where too many changes in direction make the exact position of the sample too variable and looking at the projections individually is required.

The continuous unidirectional and back‐and‐forth approaches were realigned by registering each projection with its dithered counterpart, which was due to a drift of the motors used in our setup that we could not account for otherwise. This is not a problem intrinsic in the cycloidal CT technique, but rather an issue with our specific imaging system. However, we have found that the motors drift by the same amount in each acquisition; hence it can in principle be rigorously characterized from just one dithered dataset, and this could be used to correct any subsequent continuous scans (without having to acquire a new dithered dataset each time).

The pixel‐wise strategy solves the field of view problem because the movement is limited to one pixel. However, because of this it can present ring artifacts which are not present in the other continuous approaches, and are avoided in step‐and‐shoot scanning via jittering (moving the sample a few pixels at every projection). Nonetheless, in our opinion it has the most potential out of the strategies presented here: it is time‐efficient, it does not require regridding, and it has relaxed field of view requirements.

These results confirm the potential of the cycloidal method in several implementation strategies. Despite acquiring a fraction of the frames compared to dithering (eight times fewer frames in this study), the images are similar in quality.

## 5 CONCLUSIONS

Four practical implementation strategies for cycloidal CT are presented: step‐and‐shoot, continuous unidirectional, continuous back‐and‐forth, and continuous pixel‐wise scanning. These have proven compatible with a standard table‐top x‐ray setup and are able to provide high quality images. The first three strategies provide spatial resolutions of the same order as the gold standard (dithering) and the continuous pixel‐wise images are slightly blurrier. A key strength of the continuous approaches is that they are much faster in terms of acquisition as they eliminate all dead times. Also, they have relaxed field of view requirements and provide good image quality. For these reasons, we envisage the continuous acquisition strategies to become the methods of choice for applications of cycloidal CT. These advantages, coupled with the dose reduction potential of the method, highlight the strength of cycloidal CT as an imaging method for nondestructive, high‐resolution, low‐dose applications.

## CONFLICT OF INTEREST

The authors have no conflict to disclose.
